# Editorial: Weakly supervised deep learning-based methods for brain image analysis

**DOI:** 10.3389/fninf.2022.1079240

**Published:** 2022-11-11

**Authors:** Hancan Zhu, Mingxia Liu, Zhenyu Tang, Shuai Wang

**Affiliations:** ^1^School of Mathematics Physics and Information, Shaoxing University, Shaoxing, China; ^2^Department of Radiology and BRIC, University of North Carolina at Chapel Hill, Chapel Hill, NC, United States; ^3^School of Computer Science and Engineering, Beihang University, Beijing, China; ^4^School of Mechanical, Electrical and Information Engineering, Shandong University, Weihai, China

**Keywords:** brain image analysis, deep learning, weakly supervised learning, semi-supervised learning, image segmentation, image reconstruction

In recent years, deep learning-based methods have been widely used in the fields of brain image analysis and achieved excellent performance in many tasks, including image segmentation, image reconstruction, and disease classification (Shen et al., 2017). Most of the existing deep learning-based methods rely on large-scale datasets with high-quality full annotations. However, it is usually time-consuming and requires rich expert experience to acquire such data. Moreover, because of individual differences in observer experience and understanding, large-scale and full annotated datasets may suffer from large intra- and inter-observer variability, which could hinder their application in brain image analysis. In contrast, weak yet low-cost annotations (such as coarse annotations, partial annotations, or small sample annotations) are much easier to collect than high-quality full detailed annotations. As a result, there is a strong desire for innovative deep learning-based methodologies that can efficiently learn from weakly-annotated data and achieve competitive performance compared with using full annotation data (Campanella et al., 2019).

The aim of this Research Topic is to develop effective deep learning-based methods and techniques under the supervision of different forms of weakly-annotated data in different brain image analysis tasks. When dealing with weakly-annotated data, the biggest challenge comes from “Label Ambiguity” which leads to much noise information in model learning. Nowadays, massive research has been devoted to address this problem but is still not well-studied. Fortunately, the rapid development of advanced deep learning techniques promote the continuous emergence of new solutions (Laleh et al., 2022).

The present Research Topic includes 10 original research papers on machine learning-based algorithms (most of them are deep learning methods) and applications using limited data for brain image analysis. Liu, Zhang, et al. proposed a novel co-optimization learning network (COL-Net) to train a segmentation model using limited labeled samples and unlabeled samples. Experiment results on the segmentation of penumbra tissues show that COL-Net outperforms most supervised segmentation methods. Shao et al. compared two radiomic models in predicting the progression of white matter hyperintensity (WMH) and the speed of progression using limited conventional magnetic resonance images. For rs-fMRI based autism spectrum disorder autism (ASD) diagnosis with limited data, Chu et al. developed a multi-scale graph representation learning (MGRL) framework to capture the potential complementary topology information of functional connectivity networks at different spatial scales. Based on limited functional and structural MRIs, Wang et al. proposed an adaptive multimodal neuroimage integration (AMNI) framework for automated major depressive disorder detection, with evaluation on 533 subjects with resting-state functional and T1-weighted magnetic resonance imaging (MRI) data. Liu, Wang, et al. developed a local-long range hybrid features network (LLRHNet) for medical image segmentation, which inherited the merits of the iterative aggregation mechanism and the transformer technology.

Limited by hardware conditions and other factors, high-resolution (HR) images are usually hard to obtain. Jia et al. proposed a novel super-resolution model for single 3D medical images, by using non-local low-rank tensor Tucker decomposition to exploit the non-local self-similarity prior knowledge of data. Alzheimer's disease (AD) is one of the most prevalent health threats to the elderly. Early and accurate diagnoses are significant for effective prevention and treatment of AD. Li et al. proposed to build a brain network for each subject for AD prediction and analysis, which assembled several commonly used neuroimaging data simply and reasonably, including structural MRI, diffusion-weighted imaging (DWI), and amyloid positron emission tomography (PET). Accurate labeling is essential for supervised deep learning methods. However, it is almost impossible to accurately and manually annotate thousands of images, which results in many labeling errors for most datasets. Liu, Fan, et al. proposed a local label point correction (LLPC) method to improve annotation quality for edge detection and image segmentation tasks. U-Net is widely used in medical image segmentation in recent years. Lu et al. analyzed the effects of different parts of the U-Net in the experiment of image segmentation, and proposed a more efficient architecture, called Half-UNet. Shi et al. proposed a deep learning network with subregion partition for predicting metastatic origins and EGFR/HER2 status in patients with brain metastasis.

In conclusion, these 10 papers included in this Research Topic provide new ideas on how to effectively use limited data to build reliable machine learning models for brain image analysis.

## Author contributions

All authors listed have made a substantial, direct, and intellectual contribution to the work and approved it for publication.

## Conflict of interest

The authors declare that the research was conducted in the absence of any commercial or financial relationships that could be construed as a potential conflict of interest.

## Publisher's note

All claims expressed in this article are solely those of the authors and do not necessarily represent those of their affiliated organizations, or those of the publisher, the editors and the reviewers. Any product that may be evaluated in this article, or claim that may be made by its manufacturer, is not guaranteed or endorsed by the publisher.
